# The Impact of Ischemic Stroke on Gray and White Matter Injury Correlated With Motor and Cognitive Impairments in Permanent MCAO Rats: A Multimodal MRI-Based Study

**DOI:** 10.3389/fneur.2022.834329

**Published:** 2022-03-02

**Authors:** Le Yang, Manzhong Li, Yu Zhan, Xuefeng Feng, Yun Lu, Mingcong Li, Yuming Zhuang, Jianfeng Lei, Hui Zhao

**Affiliations:** ^1^School of Traditional Chinese Medicine, Capital Medical University, Beijing, China; ^2^Beijing Key Lab of TCM Collateral Disease Theory Research, Beijing, China; ^3^Department of Pharmacy, Beijing Shijitan Hospital, Capital Medical University, Beijing, China; ^4^Beijing Key Laboratory of Bio-characteristic Profiling for Evaluation of Rational Drug Use, Beijing, China; ^5^Medical Imaging Laboratory of Core Facility Center, Capital Medical University, Beijing, China

**Keywords:** ischemic stroke, white matter, gray matter, magnetic resonance imaging, rat

## Abstract

**Background:**

Identifying the alterations of the cerebral gray and white matter is an important prerequisite for developing potential pharmacological therapy for stroke. This study aimed to assess the changes of gray and white matter after permanent middle cerebral artery occlusion (pMCAO) in rats using magnetic resonance imaging (MRI), and to correlate them with the behavior performance.

**Methods:**

Rats were subjected to pMCAO or sham surgery and reared for 30 days. Motor and cognitive function of the rats were examined by gait and Morris water maze (MWM) tests, respectively. Multimodal MRI was conducted to examine the functional and structural changes of the gray and white matter followed with luxol fast blue (LFB) staining.

**Results:**

The gait and MWM tests revealed significant motor and cognitive dysfunction in pMCAO rats, respectively. Magnetic resonance angiography presented abnormal intracranial arteries in pMCAO rats with reduced signal intensity of the anterior cerebral artery, anterior communicating cerebral artery, internal carotid artery, and increased basilar artery vessel signal compared with sham rats. Arterial spin labeling confirmed the decreased cerebral blood flow in the infarcted sensorimotor cortex and striatum. Structural T2-weighted imaging and T2 mapping showed brain atrophy and elevation of T2 value in the gray (sensorimotor cortex, striatum) and white (external capsule, internal capsule) matter of pMCAO rats. The results from diffusion tensor imaging (DTI) corresponded well with LFB staining showing reduced relative FA accompanied with increased relative AD and RD in the gray and white matter of pMCAO rats compared with sham rats. Fiber tracking derived from DTI further observed significantly reduced fiber density and length in the corresponding brain regions of pMCAO rats compared with sham rats. Specially, the DTI parameters (especially FA) in the relevant gray matter and white matter significantly correlated with the behavior performance in the gait and MWM tests.

**Conclusion:**

Collectively, the gray and white matter damages could be non-invasively monitored in pMCAO rats by multimodal MRI. DTI-derived parameters, particularly the FA, might be a good imaging index to stage gray and white matter damages associated with post-stroke motor and cognitive impairments.

## Introduction

Stroke is a leading cause of mortality and disability worldwide (1). Despite stroke mortality has been declining with effective thrombolysis, a large proportion of stroke survivors suffer permanent neurological deficits (2). It is worth noting that ischemic stroke causes not only gray matter damage defined by neuronal necrosis, but also elicits white matter injury (3). Clinical studies show that white matter accounts for half of the lesion volume in most cases of human stroke (3). Post-stroke white matter injury not only destroys the communications among different brain structures, but also causes remote gray matter dysfunction, ultimately leading to motor and cognitive impairments (4). In particular, the severity of white matter injury has been suggested to be an independent predictor of unfavorable outcomes in acute ischemic stroke patients undergoing endovascular therapy (5). Thus, numerous neuroprotective therapies aiming at cerebral gray matter injury (not specifically targeting the white matter characterized by axonal degeneration and demyelination) have failed to improve functional outcomes in clinically (6–8). Therefore, optimal therapies targeted at restoration of gray and white matter are critical to improve long-term neurological function after ischemic stroke.

Animal stroke models have played a unique role in developing new agents for stroke therapy. Since human ischemic stroke is often affected by occlusion of the middle cerebral artery (MCA), the occlusive MCA stroke models are closest to human ischemic stroke (9). To date, transient middle cerebral artery occlusion (MCAO) model with rats or mice is the widely used animal model for preclinical stroke research. As most large vessel occlusion patients have permanent vessel occlusion, recommendation of the Stroke Therapy Academic Industry Roundtable (STAIR) called for the permanent MCAO (pMCAO) model as the primary model for preclinical studies (10). Others in stroke research suggest that pMCAO model is of greater clinical relevance, and therapy beneficial in the pMCAO model may have a better chance of success in clinical trials (10). Thus, there is a need to develop reliable means that can characterize the structural alterations in gray and white matter which occur with post-stroke motor and cognitive impairments based on the pMCAO model.

In pharmacological studies, traditional histological staining techniques were used to assess the alterations of the ischemic gray and white matter. The main limitation is that it could not noninvasively monitor gray–white matter damage and repair after stroke (11). MRI provides a means to reveal the structural alterations of the post-stroke brain noninvasively and dynamically. For example, T2-weighted MRI is a favorable tool to real-time visualize the anatomical characteristics and pathological changes of the brain (12), and diffusion tensor imaging (DTI) informs on microstructural integrity of the gray and white matter (13). Hence, we implemented a multimodal MRI design to noninvasively characterize the changes in the perilesional gray and white matter in pMCAO rats, together with Morris water maze (MWM) and gait analysis. Furthermore, correlation analysis was carried out between behavior performances and DTI parameters from the gray and white matter at chronic stage of pMCAO, which could be reliably used for investigating potential stroke therapies that ameliorate ischemia-induced neurodegeneration.

## Materials and Methods

### Animals

Twenty male Sprague–Dawley rats weighing 300–320 g (aged 8 weeks) were purchased from Vital River Laboratory Animal Technology Co. Ltd. (Beijing, China). [SCXK (jing) 2016-0011] and kept under specific pathogen free (SPF) animal research center in Capital Medical University [SYXK (jing) 2018-0003] with controlled temperature (2 ± 1°C), humidity (55 ± 10%) and 12-h light/dark cycle. The experiment was carried out after 1 week of adaptive feeding. All experiments were performed according to the National Institute of Health Guide for the Care and Use of Laboratory Animals, and approved by the Ethical Committee at Capital Medical University (permit number: AEEI-2018-052). Every effort was made to minimize the number of animals used and their suffering.

### Stroke Induction and Animal Grouping

Focal cerebral ischemia was induced by permanent occlusion of the right middle cerebral artery (pMCAO) with an intraluminal filament. Briefly, 12 animals were randomly selected according to a random number list and anesthetized with isoflurane (5% for induction and 2% for maintenance) vaporized in N_2_O/O_2_ (70/30). The right common carotid artery (CCA), internal carotid artery (ICA), and external carotid artery (ECA) were carefully separated from the vagal nerves through a midline neck incision. A 4-0 monofilament nylon suture (Beijing Sunbio Biotech Co Ltd., China) was inserted from the ECA into the lumen of right ICA and advanced for about 16–18 mm until mild resistance was felt (14). After the incision was closed by a suture, rats were placed under a heating lamp until fully recovered and taken back to the standard cage with free access to food and water. The infarct volume was measured on the 31st day after pMCAO by T2-weighted imaging (T2WI). Rats with successful pMCAO induction were included into the pMCAO group (*N* = 9) with the inclusion criterion setting at a lesion volume of 100 mm^3^ (15). Two rats died in 3 days after the surgery and one rat with the infarct volume of less than 100 mm^3^ was excluded from the study. Sham-operated animals (*N* = 8) underwent the same surgical procedure without artery occlusion.

### MRI Protocols

Magnetic resonance imaging measurements were performed using a 7.0 T PharmaScan Scanner (Bruker, Germany) on the 31st day after stroke induction. Rats were initially anesthetized under 5% isoflurane mixed with N_2_O/O_2_ (70/30) and maintained under 2% isoflurane. During the MRI scan, the blood oxygen saturation and heart rate of rats were monitored, and the rectal temperature was kept at 37 ± 0.5°C. An experimenter blind to the group information performed the data analysis.

T2-weighted imaging was applied to determine the lesion volume of pMCAO rats using a fast spin-echo pulse sequence (16). The infarct region was defined by the area with image intensities higher than the mean+2 standard deviations of the intensity in the mirrored contralateral area (17). The infarct volume was calculated by multiplying total infarct area measured on each slice by the slice thickness using ImageJ software. Similarly, the ventricular volume and hemispheric volume were obtained. The hemispheric parenchymal volume was calculated by subtracting the ventricular and infarct volume from the hemispheric volume (18).

T2 mapping was conducted to assess the structural changes of gray and white matter with a multislice multiecho (MSME) sequence. Regions of interest (ROIs) were manually drawn in the bilateral gray matter (sensorimotor cortex and striatum) and white matter (internal capsule and external capsule) on coronal T2 relaxometry maps according to the Paxinos and Watson atlas. T2 values were obtained from each ROI and data were expressed as a percentage of the ipsilateral T2 values compared to the contralateral T2 values.

Three-dimensional time-of-flight magnetic resonance angiography (3D-TOF MRA) was conducted to detect the alterations of the intracranial arteries using a fast low angle shot sequence (16). The multiplanar reconstruction (MPR) and maximum intensity projection (MIP) images of MRA were generated by Paravision version 5.1 software (Bruker, Germany). The signal intensities of the bilateral anterior cerebral artery (ACA), anterior communicating cerebral artery (AcoA), anterior azygos cerebral artery (azACA), MCA, ICA, posterior cerebral artery (PCA), and basilar artery (BA) were obtained based on a previously described method (19).

Arterial spin labeling (ASL) was used to evaluate the regional cerebral blood flow (CBF) with an echo-planar imaging fluid-attenuated inversion recovery sequence (16). The heat map of ASL raw data and CBF map were obtained by Paravision version 5.1 software, and the regional CBF of the bilateral sensorimotor cortex and striatum was acquired according to our previous study (20). Data were expressed as the ipsilateral CBF relative to the contralateral.

Diffusion tensor imaging was performed to assess fiber integrity by an axial single-shot spin echo-planar imaging sequence (16). The DTI parametric maps including fractional anisotropy (FA), axial diffusivity (AD), and radial diffusivity (RD) were generated by Paravision version 5.1 software. ROIs were placed in the bilateral gray matter (sensorimotor cortex and striatum) and white matter (internal capsule and external capsule) on the DTI parametric maps to obtain corresponding DTI indices. Diffusion tensor tractography (DTT) was performed using Diffusion Toolkit and TrackVis software with the seeding areas placed on the corresponding brain regions (21). The mean fiber length and fiber density of the corresponding regions were measured. Data were presented as the ratio of ipsilateral value relative to the contralateral.

### Gait Analysis

Gait analysis was performed with DigiGait^TM^ system (Mouse Specifics Inc., America) on the 32nd day after surgery for assessing the motor function. DigiGait^TM^ contained a transparent treadmill on which animals were restricted under a polymethyl methacrylate cover and forced to walk or run at a fixed velocity. Before the experiment, rats were trained on the treadmill to make uninterrupted runs for at least 3 step-cycles at a speed of 15 cm/s. During the experiment, each rat was placed on the treadmill repeatedly at intervals of 5 min to complete three uninterrupted runs (containing at least 3 step-cycles per run) for data analyses (22). Gait parameters including stance ratio, swing ratio, brake ratio, paw area, and stride length were automatically labeled as right forelimb (RF), right hindlimb (RH), left forelimb (LF), and left hindlimb (LH). An investigator blind to the group information analyzed gait data using DigiGait Analysis15 software.

### Morris Water Maze

The Morris water maze test was performed from the 33rd to the 38th day after surgery for evaluating the learning and memory ability (23). The maze consisted of a circular tank with a transparent round platform (15 cm in diameter). The tank was divided into four virtual quadrants (quadrants I, II, III, and IV) and filled with opaque water (18–20°C).

From the 33rd to the 36th day, the platform was placed in the middle of quadrant I and 1.5 cm under the water surface for the hidden platform test. Each animal underwent four trials per day at an inter-trial interval of 60 s. For each trial, the rat was given up to 60 s to find the hidden platform. If the rat failed to find the platform within 60 s, it would be guided to the platform and kept on the platform for 10 s. The escape latency and path length by which the rat located the platform was recorded by a video tracking system (JLBehv-MWMG, Jiliang Sofware Technology Co.Ltd., Shanghai).

On the 37th day, the probe trial was performed to assess memory retention. During the probe trial, the platform was removed from the tank, and the rat was allowed to swim freely for 30 s. The percentage of escape latency and path length spent by the rat in the target quadrant (quadrant I) were calculated.

On the 38th day, rats were submitted to a platform-switched test. The submerged platform was placed in the center of quadrant II for the first trial, and then moved to quadrant III and quadrant IV for the second and third trial, respectively. For each trial, the rat was given up to 60 s to locate the hidden platform. The escape latency and path length traveled by the rat to locate the platform was analyzed. An investigator blind to the group information analyzed data.

### Immunostaining

At the end of the MWM test, rats were euthanized and transcardially perfused with 0.9% saline followed by 4% paraformaldehyde. The paraffin embedded brains were sectioned coronally to a thickness of 5 μm. Glass mounted brain sections were stained with luxol fast blue (LFB) after deparaffinization and rehydration.

### Statistical Analysis

All data were expressed as mean ± standard error of the mean (SEM). The statistical analyses were performed using the SPSS 21.0 (SPSS Inc., USA) software. Data from the hidden platform test were analyzed by two-way repeated measures ANOVA (between subject factor–surgery; within subject factor–time). The gait, probe trial, platform-switched test, and MRI data were analyzed by Student's *t*-test. The DTI data were tested for correlations with gait (left hindlimb) and MWM data using Pearson linear regression analysis. Significance was defined as *P* < 0.05.

## Results

### pMCAO Induced Motor Dysfunction in Rats

**Figure 1:**
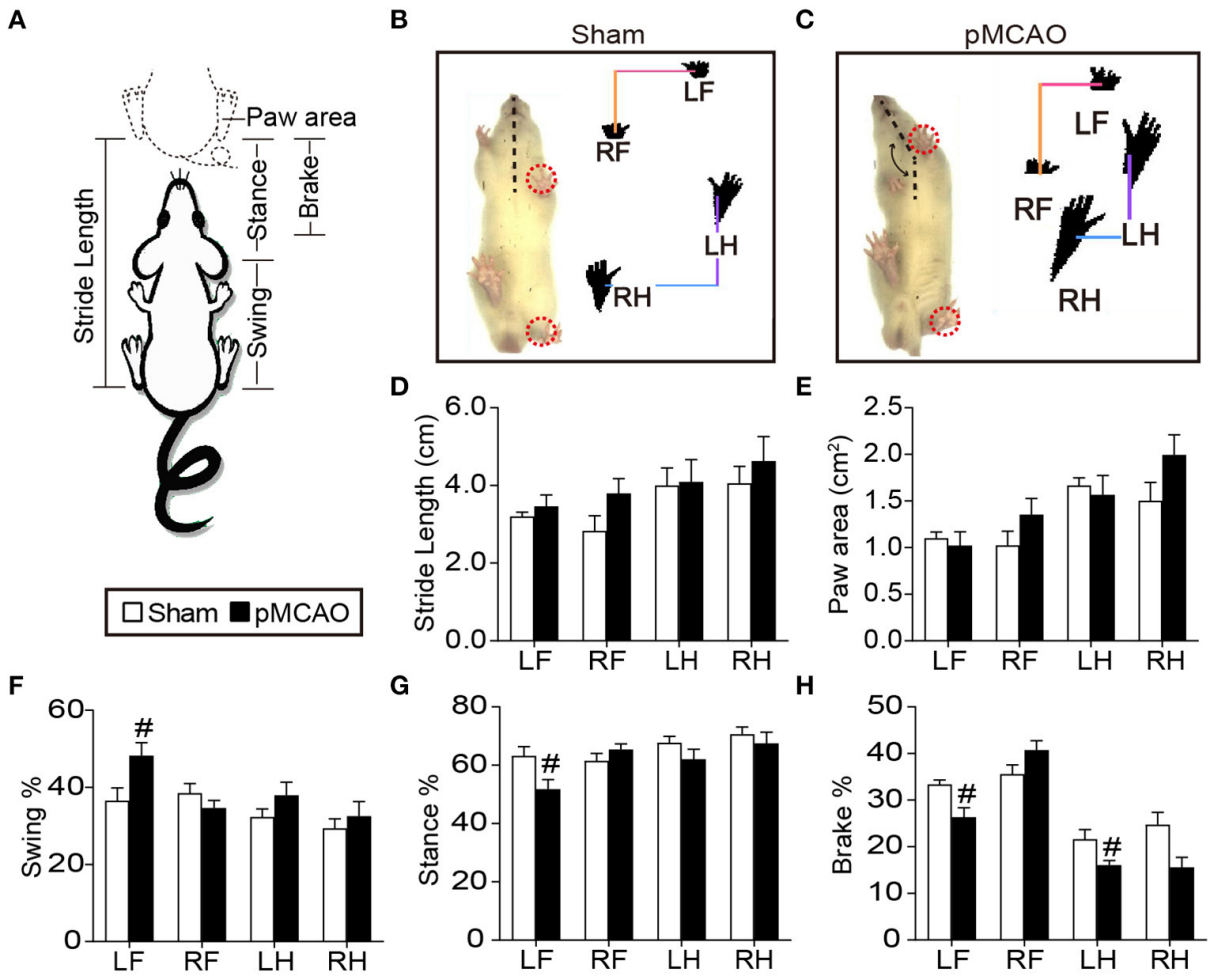
pMCAO induced motor dysfunction in rats. **(A)** Schematic diagram of gait parameters including the stride length, paw area, swing time, stance time, and brake time. **(B,C)** Typical gait pictures captured from sham and pMCAO group. **(D–H**) Quantitative analysis of the stride length, paw area, swing time, stance time, and brake time, respectively (Student's *t*-test, *N* = 8 for the sham group and *N* = 9 for the pMCAO group). ^#^*P* < 0.05, vs. sham group.

The gait analysis (Figures 1A–C) revealed that the right MCA occlusion induced significant increase in the percentage of swing time and decrease in the percentage of brake time and stance time of the left forelimb compared with sham group (*P* < 0.05, Figures 1F–H). In addition, the pMCAO rats showed reduced percentage of brake time of the left hindlimb compared with sham rats (*P* < 0.05). There was no significant difference in the paw area and stride length between the sham and pMCAO groups (Figures 1D,E). These results suggested that the occlusion of the right MCA led to remarkable motor impairments of the left limbs.

### pMCAO Caused Cognitive Decline in Rats

**Figure 2:**
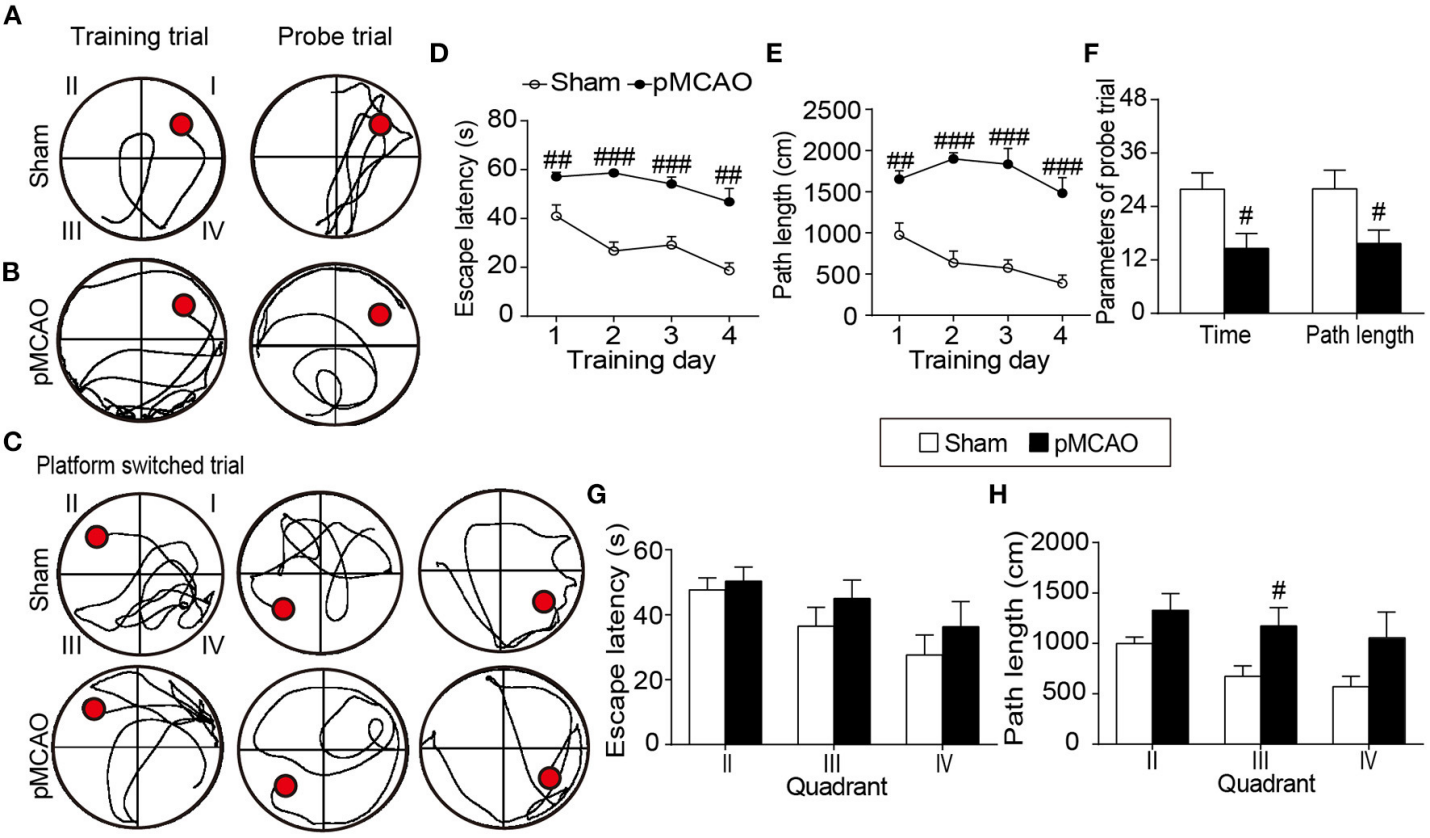
pMCAO caused cognitive decline in rats. **(A–C)** Representative swimming traces of the sham and pMCAO rats in the training trial, probe trial, and platform switched trial, respectively. **(D,E)** Quantitative data of the escape latency and path length of rats spent to locate the submerged platform during the training trial. **(F)** Quantitative analysis of the percentage time and path length of rats spent in the original quadrant in the probe trial. **(G,H)** Quantitative analysis of the escape latency and path length of rats taken to find the switched platform during the platform switched trial (Two-way repeated measures ANOVA with between subject factor-surgery and within subject factor-time for **(D)** and **(E)**. Student's *t*-test for **(F–H)**. *N* = 8 for the sham group and *N* = 9 for the pMCAO group). ^#^*P* < 0.05, ^##^*P* < 0.01, ^###^*P* < 0.001 vs. sham group.

In the hidden platform test, repeated measures of ANOVA showed significant main effects of time and group on the escape latency [*F*_time(3, 21)_ = 17.214, *F*_group(1, 7)_ = 69.067, *P* < 0.001], and path length [*F*_time(3, 21)_ = 4.978, *F*_group(1, 7)_ = 47.673, *P* < 0.01–0.001, Figure 2A], indicating all the rats showed enhanced spatial learning over training days. Group comparisons revealed that pMCAO rats took longer escape latency and path length to locate the submerged platform from the 1st to the 4th training day compared with the sham rats (*P* < 0.01–0.001, Figures 2D,E), suggesting impaired spatial learning ability after pMCAO. In the probe trial, pMCAO rats spent less time and covered shorter path length in the target quadrant compared with the sham rats (*P* < 0.05, Figures 2B,F), demonstrating disrupted memory retention after pMCAO. In the platform-switched trial, pMCAO rats took longer path length to locate the switched platform in the quadrant III than the sham rats (*P* < 0.05, Figures 2C,G,H), indicating impaired working memory after pMCAO.

### pMCAO Resulted in Impaired Cerebrovascular Hemodynamics in Rats

**Figure 3:**
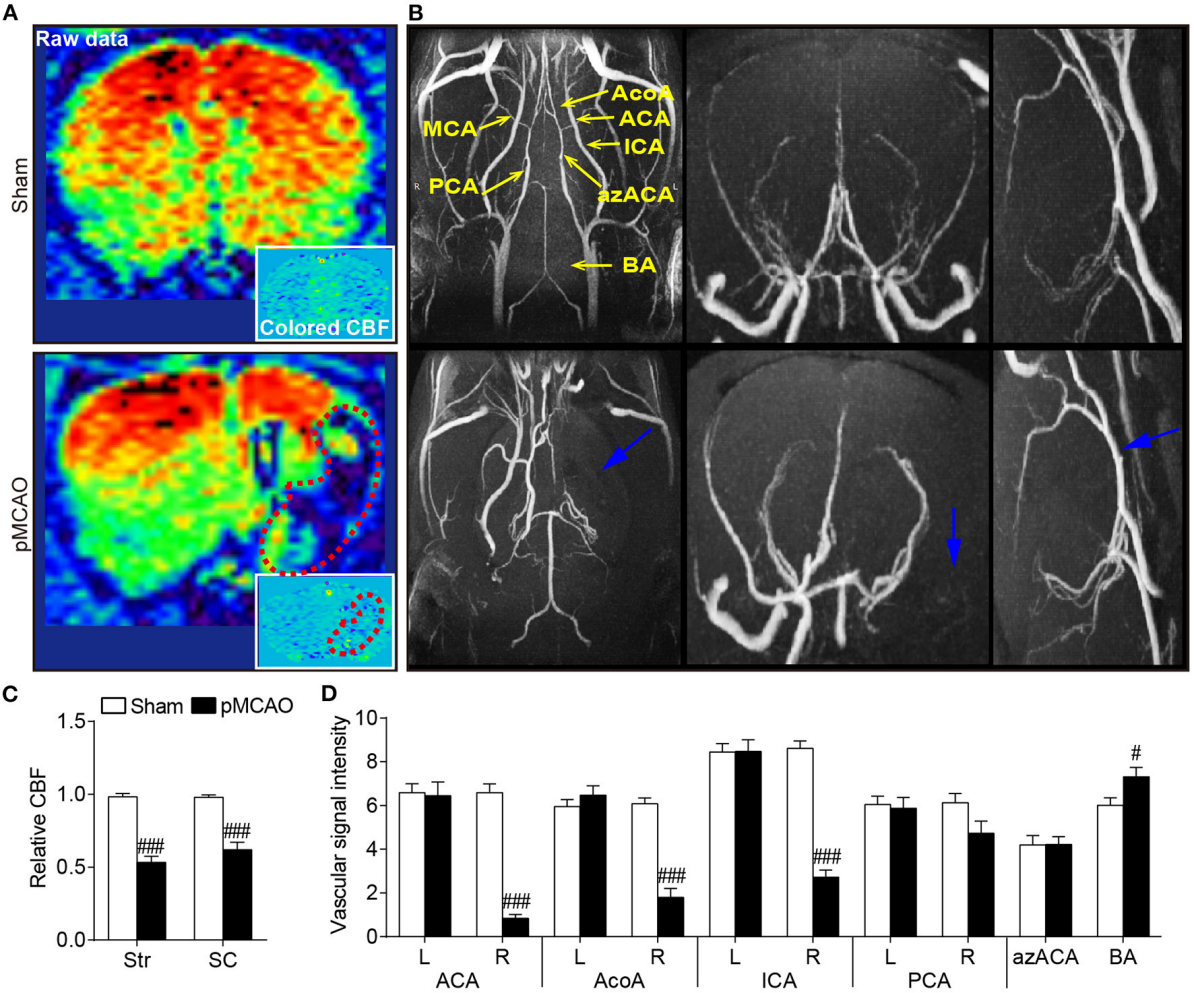
pMCAO resulted in impaired cerebrovascular hemodynamics in rats. **(A)** Representative coronal raw data and colored CBF maps from the sham and pMCAO rats. **(B)** Typical axial, coronal, and sagittal MIP images of the sham and pMCAO rats. **(C)** Quantitative data of relative CBF in the sensorimotor cortex (SC) and striatum (Str). **(D)** Quantitative analysis of the vascular signal intensity from the bilateral anterior cerebral artery (ACA), anterior communicating cerebral artery (AcoA), internal carotid artery (ICA), posterior cerebral artery (PCA), anterior azygos cerebral artery (azACA), and basilar artery (BA) (Student's *t*-test, *N* = 8 for the sham group and *N* = 9 for the pMCAO group). ^#^*P* < 0.05, ^###^*P* < 0.001 vs. sham group.

The angiographic MIP maps presented the connections and morphologies of the intraluminal arteries including the AcoA, azACA, ACA, MCA, PCA, ICA, and BA (Figure 3B). In the pMCAO rats, the signal intensity in the right MCA starting at its origin through the distal parts was totally absent (blue arrows), suggesting the successful occlusion of the right MCA. The signal intensities of the ipsilateral ACA, AcoA, and ICA were significantly reduced in the pMCAO rats compared with the sham rats (*P* < 0.001, Figure 3D). Additionally, the pMCAO rats exhibited higher signal intensity in the BA than the sham rats (*P* < 0.05). Moreover, the ASL results showed that the relative CBF of the ipsilateral sensorimotor cortex and striatum were markedly decreased in the pMCAO rats compared with the sham rats (*P* < 0.001, Figures 3A,C), indicating that the deleterious alterations of the collateral vessels further resulted in inadequate blood supply to the corresponding brain regions.

### pMCAO Induced Brain Atrophy and Structural Injury of the Gray and White Matter in Rats

**Figure 4:**
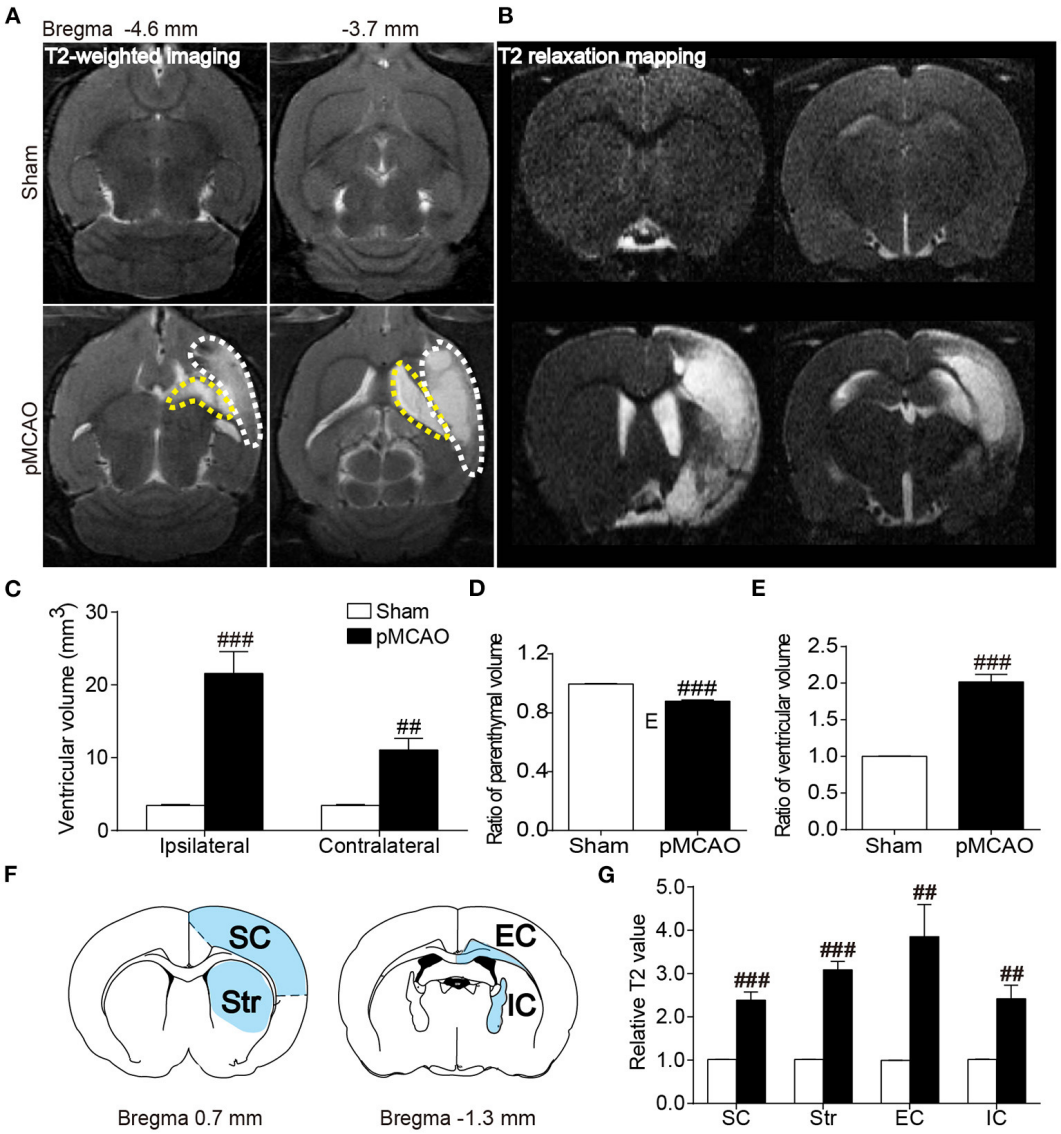
pMCAO induced brain atrophy and structural injury of the gray and white matter in rats. **(A,B)** Representative axial T2WI and coronal T2 relaxation mapping images of the sham and pMCAO rats. **(C–E)** Quantitative analysis of the ventricular volume, ratio of parenchymal volume, and ratio of ventricular volume, respectively. **(F)** Brain atlas showing the regions of interest including sensorimotor cortex (SC), striatum (Str), external capsule (EC), and internal capsule (IC). **(G)** Quantitative analysis of the relative T2 value (Student's *t*-test, *N* = 8 for the sham group and *N* = 9 for the pMCAO group). ^##^*P* < 0.01, ^###^*P* < 0.001 vs. sham group.

Axial T2WI images exhibited obvious hyperintensity in the ipsilateral hemisphere accompanied with remarkably enlarged bilateral ventricles (Figure 4A). Quantitative data showed that the bilateral ventricular volumes were increased in the pMCAO rats compared with the sham rats (*P* < 0.01–0.001, Figures 4C,E). Moreover, rats in the pMCAO group had smaller parenchymal volume than that of sham rats (*P* < 0.001, Figure 4D), indicating severe brain atrophy following stroke. The relative T2 values of the ipsilateral gray matter (sensorimotor cortex, striatum) and white matter (external capsule, internal capsule) were significantly elevated in pMCAO rats compared with sham rats (*P* < 0.01–0.001, Figures 4B,F,G).

### pMCAO Induced Microstructural Damages of the Gray and White Matter in Rats

**Figure 5:**
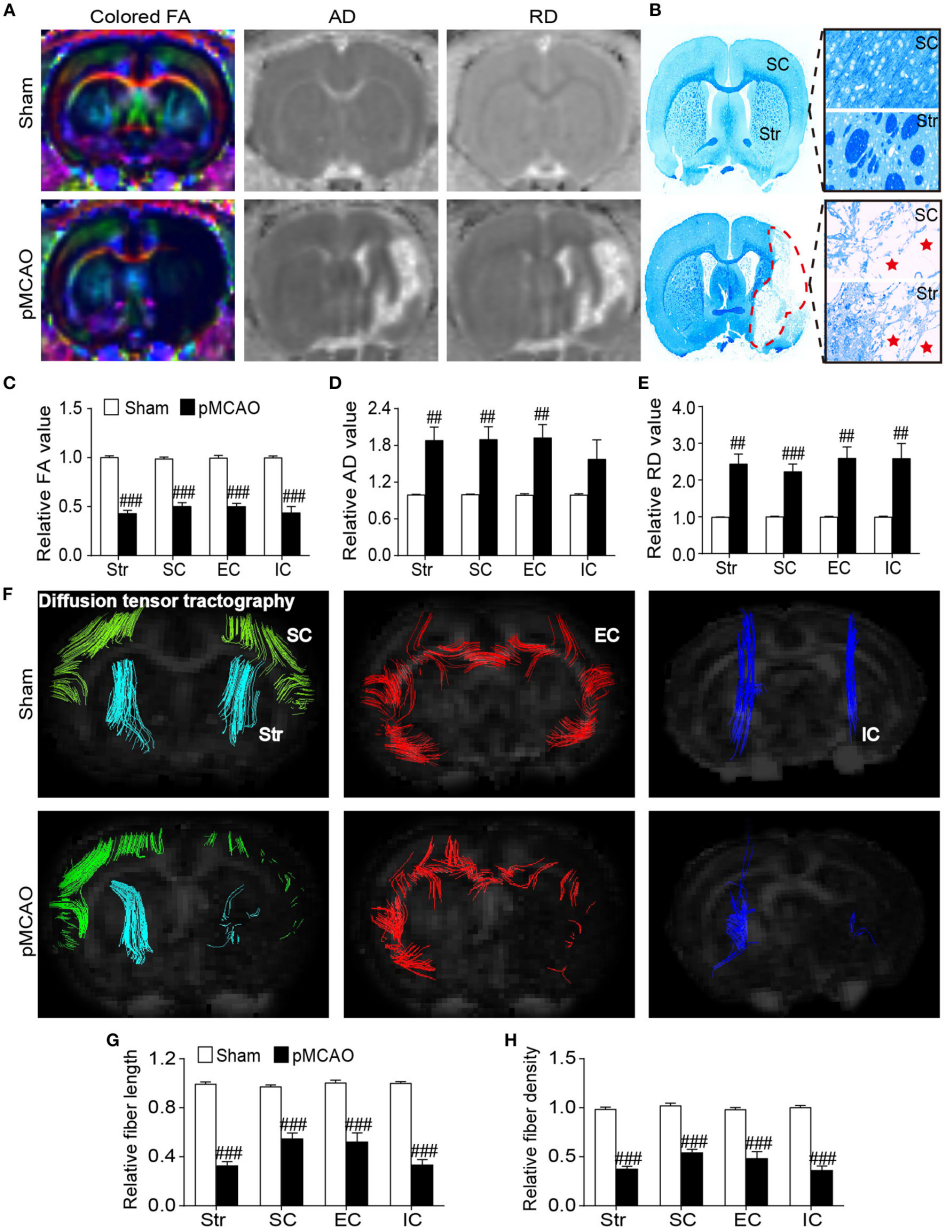
pMCAO induced microstructural damages of the cerebral gray and white matter in rats. **(A)** Representative colored FA, AD, and RD images of the sham and pMCAO rats. **(B)** Typical LFB staining images of the sham and pMCAO rats. **(C–E)** Quantitative analysis of the relative FA, AD, and RD, respectively. **(F)** Diffusion fiber tracking with the seed placed on the bilateral sensorimotor cortex (SC), striatum (Str), external capsule (EC), and internal capsule (IC). **(G,H)** Quantitative analysis of the relative fiber length and fiber density, respectively (Student's *t*-test, *N* = 8 for the sham group and *N* = 9 for the pMCAO group). ^##^*P* < 0.01, ^###^*P* < 0.001 vs. sham group.

Diffused tensor imaging results showed significantly reduced relative FA accompanied with elevated relative AD and RD in the ipsilateral sensorimotor cortex, striatum, and external capsule of the pMCAO rats compared with the sham rats (*P* < 0.01–0.001, Figures 5A,C–E). Besides, the decreased relative FA and increased relative RD were also observed in the internal capsule of the pMCAO rats compared with sham rats (*P* < 0.01–0.001). The LFB staining correlated well with DTI findings showing severe axonal disorganization after pMCAO (Figure 5B). Fiber tracking further validated the axonal damages following pMCAO as revealed by remarkably decreased relative fiber density and fiber length in the corresponding brain regions of the pMCAO rats in comparison with the sham rats (*P* < 0.01–0.001, Figures 5F–H).

### pMCAO Induced Motor Dysfunctions Were Correlated to Gray and White Matter Damages in Rats

**Figure 6:**
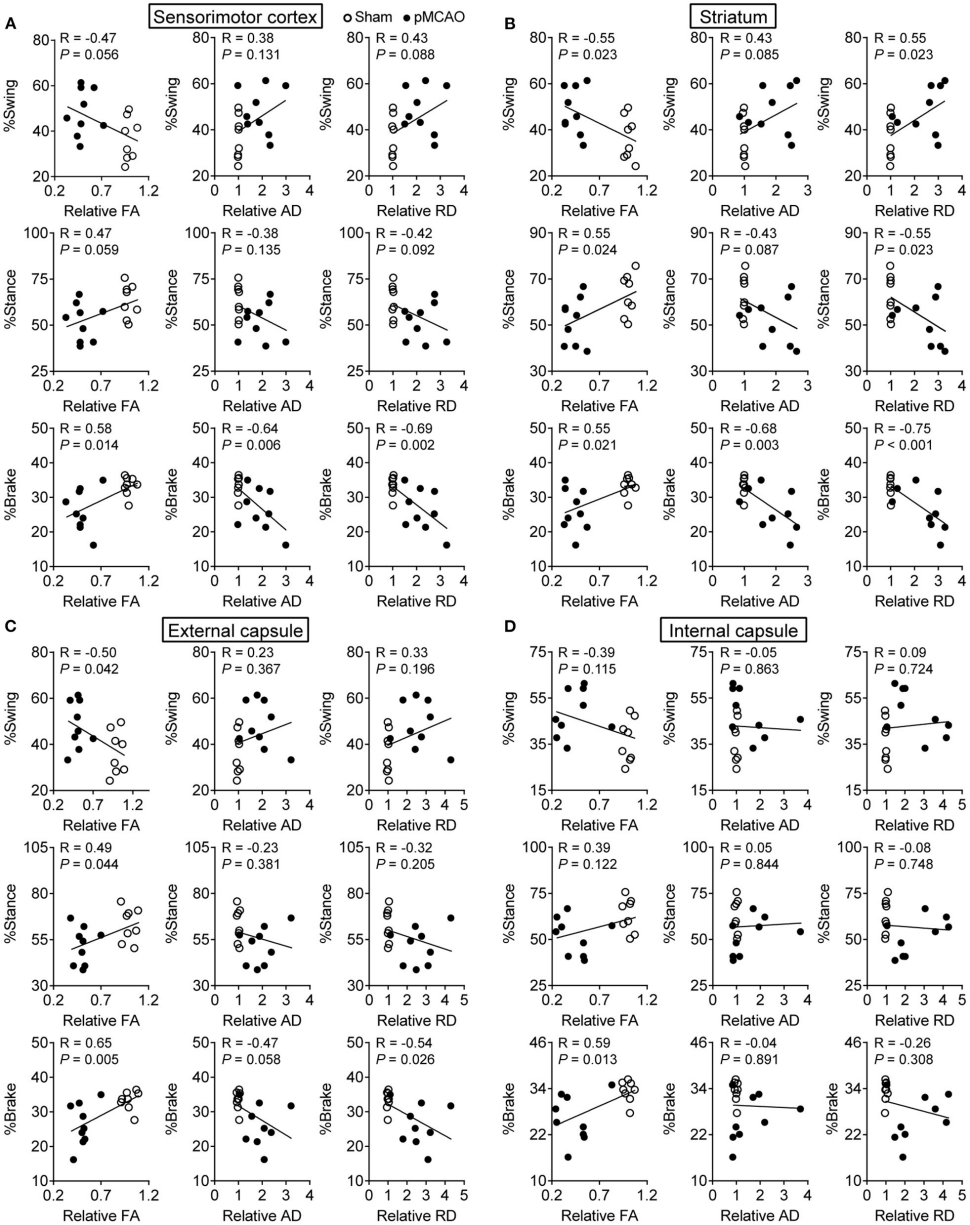
pMCAO induced motor dysfunctions were correlated to gray and white matter damages in rats. Correlational analysis between the gait data and DTI parameters obtained from the **(A)** sensorimotor cortex, **(B)** striatum, **(C)** external capsule, and **(D)** internal capsule.

The Pearson linear regression analysis revealed significant correlations between the percentage of brake time and DTI index in the cerebral gray matter including the sensorimotor cortex and striatum (*P* < 0.05–0.001, Figures 6A,B). The relative FA and RD of the striatum were also related to the percentage of swing and stance time (*P* < 0.05). For the cerebral white matter, there were notable correlations between the relative FA in the external capsule and gait parameters (*P* < 0.05–0.01, Figure 6C). The relative RD in the external capsule and relative FA in the internal capsule were also significantly correlated with the percentage of brake time (*P* < 0.05, Figure 6D). The significant correlations between the gait and DTI parameters were concluded in Table 1. These results suggested that the structural damages of the cerebral gray and white matter might contribute to post-stroke motor dysfunctions.

**Table 1:** The summary of significant correlations between the gait and DTI index.

**Index**	**Sensorimotor cortex**	**Striatum**	**External capsule**	**Internal capsule**
%Swing	–	FA, RD	FA	–
%Stance	–	FA, RD	FA	–
%Brake	FA, AD, RD	FA, AD, RD	FA, RD	FA

### pMCAO Induced Cognitive Impairments Were Correlated to Gray and White Matter Injuries in Rats

**Figure 7:**
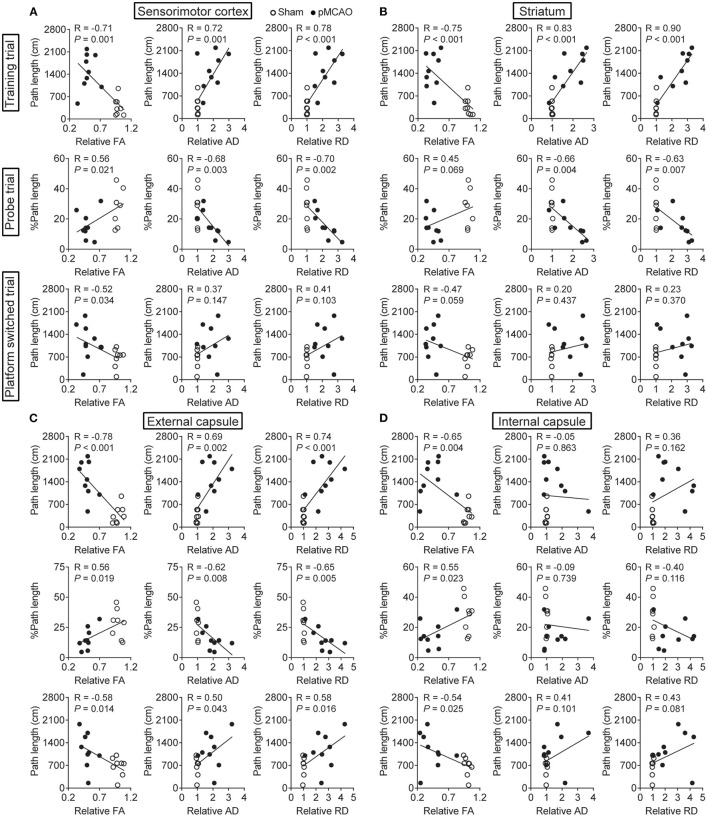
pMCAO induced cognitive impairments were correlated to gray and white matter injuries in rats. Correlational analysis between the MWM data and DTI parameters obtained from the **(A)** sensorimotor cortex, **(B)** striatum, **(C)** external capsule, and **(D)** internal capsule.

For the cerebral gray matter, the relative FA, AD, and RD of the sensorimotor cortex and striatum were significantly correlated with the path length in the training trial (*P* < 0.01–0.001, Figures 7A,B). There were also remarkable correlations between the percentage of path length in the probe trial and the relative AD and RD of the sensorimotor cortex and striatum (*P* < 0.05–0.01). Besides, relative FA in the sensorimotor cortex also related to the percentage of path length in the probe trial and path length in the platform-switched trial (*P* < 0.05). For the white matter, all the DTI index of the external capsule and the relative FA of the internal capsule were significantly correlated with all the WMW data (*P* < 0.05–0.001, Figures 7C,D). The significant correlations between the MWM and DTI parameters are concluded in Table 2. These data indicated that poststroke cognitive impairments might be attributed to the structural damages of the cerebral gray and white matter.

**Table 2:** The summary of significant correlations between the MWM and DTI index.

**Index**	**Sensorimotor cortex**	**Striatum**	**External capsule**	**Internal capsule**
Training trial	FA, AD, RD	FA, AD, RD	FA, AD, RD	FA
Probe trial	FA, AD, RD	AD, RD	FA, AD, RD	FA
Platform switched trial	FA	–	FA, AD, RD	FA

## Discussion

Cerebral ischemia is one of the most common causes of adult long-term disability, and therapeutic approaches aimed at boosting rehabilitative processes is of great concern (3). The pMCAO model is more convenient for investigating the effects of therapeutic approaches on functional recovery and structural plasticity of the gray and white matter in chronic phase after ischemic stroke (24). According to our multimodal MRI, the present study found that the structural impairments of the cerebral gray and white matter were related to motor and cognitive dysfunction in pMCAO rats on the 30th day after stroke. It was worth noting that MRI parameters, particularly the DTI parameter FA, showed significant and strong correlation with the alterations of gait and learning-memory behavior. This indicated that the DTI parameter might be an important evaluation index for monitoring the behavioral impairment.

Ischemic stroke is most caused by stenosis or occlusion of cerebral arteries. MRA provides a non-invasive technique that allows spatiotemporally observing the changes of intracranial arteries (25). In the present study, we applied MRA to detect the brain arteries involving the circle of Willis which is the major route for the redistribution of cerebral blood flow after ischemic stroke (26). Our results showed that the unilateral MCA occlusion resulted in enhanced signal intensity of the BA, indicating that vertebrobasilar artery system might redistribute collateral flow *via* the circle of Willis in a compensatory manner (27). However, this compensation was insufficient. ASL imaging provided direct evidence showing the significantly reduced blood flow in the brain areas mainly supplied by the middle cerebral artery, especially in the sensorimotor cortex and striatum.

The continuous hypoperfusion could induce extensive tissue loss. Currently, serial T2WI images showed a large scale of infarction in the MCA-supply areas of the ipsilateral hemisphere and detected brain atrophy as evidenced by remarkably enlarged bilateral ventricles and reduced ipsilateral parenchyma volume in pMCAO rats, which was consistent with previous studies (28, 29). Specifically, T2 mapping detected significantly prolonged T2 relaxation time in both the gray matter (sensorimotor cortex, striatum) and white matter (external capsule, internal capsule). Based on this, this study strongly supported that pMCAO rats showed impairment of both gray matter and white matter.

To address the effects of pMCAO on microstructural changes in the gray and white matter, we further performed DTI analysis. DTI is considered as a sensitive tool to monitor the microstructural integrity of gray and white matter (13). FA is the most used parameter calculated from DTI that characterizes the spatial density, distribution, and connection of the nerve fibers (30). AD and RD are highly specific for characterizing the alterations of axon and myelin sheath, respectively (31). In this study, decreased FA accompanied with increased AD and RD were detected in both the ischemic gray matter (sensorimotor cortex, striatum) and white matter (external capsule, internal capsule) following stroke. Generally, decreased FA in the lesioned areas indicates demyelination and axonal injury after stroke, and increased AD and RD may result from widespread axonal disorganization and myelin degeneration, respectively (32). Moreover, the DTI-derived fiber tracking demonstrated that the ipsilateral fiber bundles were disorganized accompanied with significantly reduced fiber density and length in the corresponding regions. Correspondingly, LFB staining provided direct evidence showing the pathologic demyelination and axonal breakdown in the white and gray matter following stroke.

Due to the damage of neurons and descending fibers, most stroke survivors suffer from persistent neurological deficits with limited recovery of function (33). Motor dysfunction, characterized by decreased muscle strength and motor coordination, is one of the most common clinical manifestations in stroke. It is well-known that sensorimotor cortex and striatum play a major role in regulating muscle tension and coordinating complex movement (34). While ischemic stroke also elicits white matter injury, increasing evidence suggested that disorganized white matter tracts and the resultant loss of connectivity to the cortical regions greatly contributed to poor motor performance (35). Particularly, internal capsule is a highly concentrated part of motor and sensory conduction fibers in the corticospinal tract (36). The integrity of internal capsule is closely related to the prognosis of motor function in stroke patients (37). Moreover, the external capsule partially connects with the internal capsule, and the external capsule impairment was closely related to the lower limb spasm of stroke patients (38).

Previous studies reported that gait changes observed in pMCAO rats were more similar with changes in humans following cerebral ischemia (24). Automated gait analysis is a novel method for monitoring motor deficit in animal models of stroke (13). With the information obtained from the Digi-automated gait analysis, our results were in accordance with previous studies showing that permanent right MCA occlusion induced notable left hindlimb impairments as revealed by elevated swing time accompanied with reduced stance and brake time (24).

Clinical reports showed that white matter injury detected by MRI is a stronger predictor of serious motor function symptoms in stroke patients (39). Similarly, we found there were significant correlations between the gait and DTI parameters. Especially FA in the gray matter (sensorimotor cortex, striatum) and white matter (internal capsule, external capsule) were obviously correlated with functional deficits of gait on the 31st day after pMCAO. Overall, these findings suggested that DTI parameters from gray and white matter might be appropriate noninvasive imaging biomarkers for the long-term evaluation of locomotor function deficits in pMCAO rats.

In addition to limb motor function, cognitive impairment is also a common complication following an ischemic stroke (40). In the present study, the MWM results showed significant spatial learning and memory impairments in pMCAO rats, which agreed with previous study (41). Previous reports indicated that the sensorimotor cortex and striatum were closely linked to poststroke learning and memory impairments (42–44). In addition to gray matter, damages to the white matter fibers played critical roles in the attention and executive function (45). Specially, lesion in the bilateral external capsule involved in the cognitive impairment following status epilepticus (46, 47). We analyzed the correlation between MWM and DTI parameters. Similarly, the results showed remarkable correlations between the changes in MWM data and changes in DTI parameters, indicating that DTI can provide an effective method to detect learning and memory impairment in pMCAO rats.

In conclusion, the present study detected varying degrees of structural damages in the gray and white matter in pMCAO rats by multimodal MRI. Specially, the DTI parameters significantly correlated with the behavior data from gait and MWM analysis, suggesting that MRI-derived parameters could be favorable predictors of post-stroke motor and cognitive impairments. Our findings might bring some new clues for identifying gray and white matter damages associated with the decline of both motor and cognitive functions, which is imperative to evaluate the efficacy of any potential pharmacological therapy in a preclinical phase.

## Data Availability Statement

The raw data supporting the conclusions of this article will be made available by the authors, without undue reservation.

## Ethics Statement

The animal study was reviewed and approved by Capital Medical University.

## Author Contributions

LY and MaL performed experiments, analyzed data, and drafted the manuscript. YZha and XF carried out animal experiments. LY, MiL, and JL conducted magnetic resonance imaging experiments. YZhu contributed to immunostaining. HZ designed the study, supervised the whole project, and reviewed the manuscript. All authors contributed to the article and approved the submitted version.

## Funding

This work was supported by the National Natural Science Foundation of China (Grant nos. 82174471 and 81774381) and the Beijing Municipal Natural Science Foundation (Grant no. 7212161).

## Conflict of Interest

The authors declare that the research was conducted in the absence of any commercial or financial relationships that could be construed as a potential conflict of interest.

## Publisher's Note

All claims expressed in this article are solely those of the authors and do not necessarily represent those of their affiliated organizations, or those of the publisher, the editors and the reviewers. Any product that may be evaluated in this article, or claim that may be made by its manufacturer, is not guaranteed or endorsed by the publisher.
